# Outcomes and interventions in patients transported to hospital with ongoing CPR after out-of-hospital cardiac arrest – An observational study

**DOI:** 10.1016/j.resplu.2021.100170

**Published:** 2021-10-16

**Authors:** S. Schmidbauer, EJ. Yates, C. Andréll, D. Bergström, H. Olson, GD. Perkins, H. Friberg

**Affiliations:** a Center for Cardiac Arrest, Department of Clinical Sciences Lund, Lund University, Lund, Sweden; b Department of Intensive and Perioperative Care, Skåne University Hospital, Malmö, Sweden; c The Dudley Group NHS Foundation Trust, West Midlands DY1 2HQ, UK; d Team CPR, Practicum Clinical Skills Centre, Office of Medical Services, Region Skåne, Sweden; e Warwick Clinical Trials Unit and University Hospitals Birmingham, University of Warwick, Coventry CV4 7AL, UK

**Keywords:** Transport with ongoing CPR, Transport with ongoing resuscitation, Reversible causes of cardiac arrest, Outcome

## Abstract

**Introduction:**

The main objective was to present characteristics and outcome of patients without sustained field return of spontaneous circulation (ROSC) transported to hospital with ongoing cardiopulmonary resuscitation (CPR). Our secondary objectives were to investigate hospital-based interventions and the performance of the universal Termination of Resuscitation-rule (uTOR).

**Methods:**

In this retrospective observational cohort study, out-of-hospital cardiac arrest (OHCA) patients arriving to the emergency department of a university hospital in Sweden during a six-year period (2010–2015) were identified using a prospectively recorded hospital-based registry. Additional data were retrieved from medical records and from the Swedish cardiopulmonary resuscitation registry.

**Results:**

Among 409 patients transported with ongoing CPR, 7 survived to hospital discharge (1.7%). Hospital-based interventions against a suspected cause of arrest were attempted during ongoing resuscitation in 34 patients (8.3%), of whom 3 survived to hospital discharge. The remaining 4 survivors had spontaneous in-hospital ROSC. Survivors presented with either a shockable rhythm (*n* = 4) or pulseless electrical activity (*n* = 3). The uTOR identified non-survivors with a positive predictive value (PPV) of 98.4% and a specificity of 71.4% for termination.

**Conclusion:**

Survival after OHCA where sustained prehospital ROSC is not achieved is rare and available in-hospital interventions are rarely utilised. No patient with asystole as the first recorded rhythm survived. The uTOR identified non-survivors with a PPV of 98.4% but showed poor specificity.

## Introduction

Approximately 2 in 5 patients with prehospital return of spontaneous circulation (ROSC) after out-of-hospital cardiac arrest (OHCA) survive to hospital discharge.1., 2. For patients with refractory OHCA in the field, however, outcomes are poor and management of these patients constitutes a big challenge for emergency medical services (EMS).3., 4. Whether these patients would benefit from transportation to hospital with ongoing cardiopulmonary resuscitation (CPR) or remain for continued CPR in the field is a matter of debate.^5^ Several clinical decision rules have been proposed to help identify patients who might benefit from being transferred to hospital versus those who would not. The universal termination of resuscitation rule (uTOR) is widely used and the best validated decision rule to date.6., 7., 8. The uTOR criteria include no shocks administered, unwitnessed by the EMS and no ROSC.^9^

Since the advent of automated chest compression devices, transport to hospital with ongoing resuscitation has become more practically feasible, but for such transport to be meaningful, the resources of receiving hospitals must add value for the patient. A high degree of automated chest compression device utilisation after OHCA^10^ is seen in the Skåne region in southern Sweden, and regional guidelines used to recommend prompt transportation to hospital for a majority of OHCA patients, irrespective of whether prehospital ROSC was achieved or not.^11^

In this setting, where the EMS have long-standing experience of automated chest compression devices and of transporting OHCA patients with ongoing CPR, our main objective was to examine the characteristics and outcome of transported patients. Secondary objectives were to study hospital-based interventions and the proportion of transported patients who would have met the uTOR criteria for termination of CPR in the field.

### Methods

#### Study design

This was a single-centre retrospective cohort study including consecutive patients arriving in the emergency department (ED) after OHCA between January 2010 through December 2015. Patients were identified using a local hospital-based quality registry.

#### Study setting

Skåne University Hospital in Lund, Sweden, is a tertiary referral and teaching hospital with a primary catchment population of about 330 000 and around-the-clock cardiac catheterisation capabilities seven days a week, supporting the entire region of Skåne with a population of 1.3 million during non-office hours. Although serving as the regional ECMO-centre, no routine algorithm for extracorporeal cardiopulmonary resuscitation (eCPR) was in place during the study period.

Ambulances are staffed by a crew of 2 of whom at least one is a registered nurse with specialist training. At the time, specialised nurses constituted 61% of the regional EMS staff, emergency medical technicians (EMTs) 25% and registered nurses without specialist training 14%.^12^ Ambulance personnel have around-the-clock access to an over-the-phone physician consultant.

Prehospital advanced life support is provided in accordance with a regional adaptation of the Advanced Life Support (ALS) algorithm.^13^ This includes drug administration (adrenaline, amiodarone and naloxone), the use of a manual external defibrillator and advanced airway management, predominately using supraglottic devices. After ALS initiation, prompt transport to hospital was encouraged for all patients throughout the study period - regardless of ROSC or initial rhythm. All ambulances carry an automated chest compression device (LUCAS™, Stryker Medical/Jolife AB, Lund, Sweden) and guidelines advocate their use in all patients with cardiac arrest.^11^ Ambulance personnel were legally authorised to terminate resuscitation efforts at their own discretion, typically in adult patients with continuous asystole and CPR for 30 min. Guidelines for termination of resuscitation were in place throughout the study period.

For suspected OHCA, two ambulances are routinely dispatched and, in addition, fire fighters are used as first responders if the dispatcher estimates a time gain. Fire fighters have Basic Life Support (BLS) training and carry automatic external defibrillators (AEDs).

### Data

The in-hospital cardiac arrest team responds to in-hospital emergencies, including all cases of OHCA arriving to the hospital, regardless of whether ROSC has been achieved prior to arrival or not. All team responses along with baseline and treatment data are logged in a local quality registry using an Utstein-style template.^14^

In the present study, patients arriving in the ED after OHCA from January 1, 2010 through December 31, 2015 were identified. Patients with cardiac arrest in the ambulance were excluded. Using automatic matching on social security numbers, additional background and prehospital treatment data were retrieved from the Swedish Registry of Cardiopulmonary Registration (SRCR). For cases with incomplete social security numbers in either registry, potential matches were identified with a script-based search on overlapping data including age, sex, date of arrest, witnessed status and initial rhythm. Records were then scrutinised manually for consistency before being added to the study database.

To gather detailed data on hospital-level treatment and ROSC status, individual medical records were screened using pre-defined criteria. Interventions against possible causes of cardiac arrest were classified as per the original 4 H and 4 T-classification^13^ and categorized as presented in table S1. Any therapy identified in medical notes from the ED that fell outside this classification and the ALS algorithm was classified as “supportive” and noted as free text. A post-hoc categorisation is presented in table S2. Interventions and supportive therapies were classified only if initiated during ongoing CPR.

Sustained ROSC was defined as 20 consecutive minutes or more of spontaneous circulation without the need for chest compressions. For cases where explicit, time-stamped records were lacking, sustained ROSC was assumed for all cases without evidence of rearrest. For cases with ROSC and evidence of at least one re-arrest, the ROSC period was classified as either sustained or non-sustained based on clinical judgement from available data or left as missing data if uncertain. The location of ROSC episodes was classified depending on where ROSC first occurred (i.e. a patient with sustained ROSC 10 minutes prior to hospital arrival was classified as prehospital sustained ROSC). Due to sparse prehospital records, exact timing of prehospital ROSC episodes (i.e. before or after commencement of transport) could not always be determined. Any ROSC prior to hospital arrival was therefore used in the determination of uTOR-status.

### Statistical analysis

Data analysis was performed using R (version 4.0.3). Continuous variables were graphically assessed for normality and presented as mean +/- SD or median (IQR) as appropriate. Parametric between-group comparisons were performed using Student’s *t*-test, whereas the Kruskal-Wallis test was used for corresponding non-parametric comparisons. Dichotomous data are presented as absolute and relative frequencies, and between-group comparisons were performed using either Pearson’s chi-squared test or – when the expected count of any cell was less than 5 – Fisher’s exact test. Relative frequencies were estimated with the total *n* of each respective group as the denominator regardless of any missing values. Confidence intervals for predictive values are the standard logit confidence intervals given by Mercaldo et al.^15^ Best and worst case sensitivity analyses were performed to quantify the effect of missing data on uTOR-performance.

## Results

**Fig. 1:**
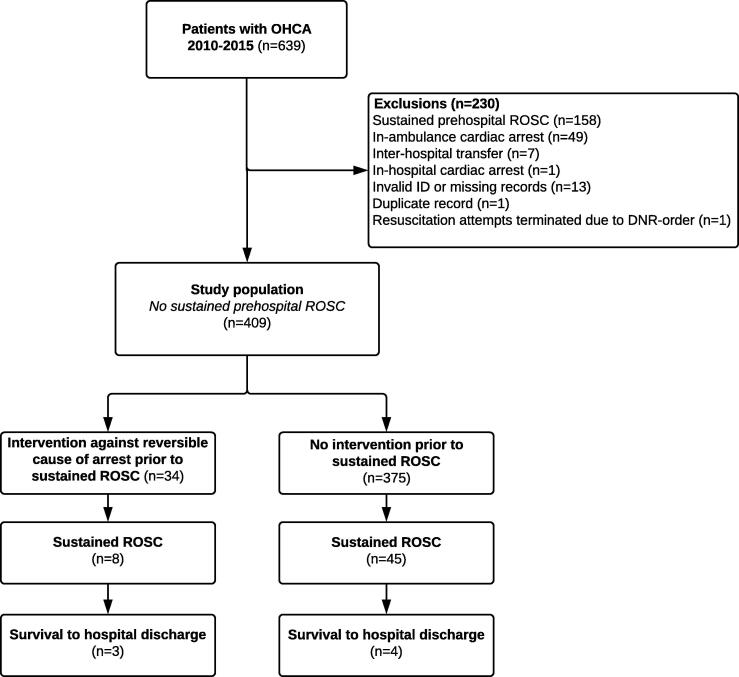
Flow of patients. OHCA: Out-of-hospital cardiac arrest. ROSC: Return of spontaneous circulation.

During the six-year study period, 639 patients arriving in the ED after OHCA were identified in the local cardiac arrest quality registry. Of those, 158 had achieved sustained ROSC in the prehospital setting (table S3) and 72 were excluded for other reasons, leaving 409 patients to constitute the study population (Fig. 1).

### Baseline characteristics in relation to survival to hospital discharge

Among patients arriving with no sustained ROSC, 7 of 409 patients (1.7%) survived to hospital discharge (Table 1). Sustained ROSC was achieved in the ED in 53 of 409 patients (13.0%) of whom all but one (*n* = 52) were admitted to a hospital ward; 47 to intensive care. Survivors (*n* = 7) had a median time to sustained ROSC of 30 minutes (IQR 27.5–55 min). All 7 survivors had a witnessed arrest, a majority (4 of 7) had a shockable initial rhythm and the remaining 3 presented with PEA. No patient with initial asystole survived to hospital discharge. Three of seven survivors had non-sustained ROSC prior to hospital arrival and a majority of survivors (4 of 7) suffered cardiac arrest at a public location.

**Table 1:** Baseline characteristics by survival to hospital discharge.

	Survival to hospital discharge			
	All patients *N* = 409	Yes *N* = 7	No *N* = 402	*P* value
**Age (years)**	68.3 (16.3)	68.0 (16.7)	68.3 (16.3)	0.963
**Sex: Male**	281 (68.7%)	4 (57.1%)	277 (68.9%)	0.683
**Initial rhythm:**				0.051
Shockable	133 (32.5%)	4 (57.1%)	129 (32.1%)	
Asystole	181 (44.3%)	0 (0.00%)	181 (45.0%)	
PEA	90 (22.0%)	3 (42.9%)	87 (21.6%)	
Unknown, no shocks delivered	4 (0.98%)	0 (0.00%)	4 (1.00%)	
Missing	1 (0.24%)	0 (0.00%)	1 (0.25%)	
**Defibrillation performed**	183 (44.7%)	3 (42.9%)	180 (44.8%)	1.000
Missing	12 (2.93%)	1 (14.3%)	11 (2.74%)	
**Witnessed**	286 (69.9%)	7 (100%)	279 (69.4%)	0.109
Missing	1 (0.24%)	0 (0.00%)	1 (0.25%)	
**EMS witnessed**	38 (9.29%)	0 (0.00%)	38 (9.45%)	1.000
Missing	78 (19.1%)	3 (42.9%)	75 (18.7%)	
**Presumed cardiac cause of arrest**	191 (46.7%)	2 (28.6%)	189 (47.0%)	1.000
Missing	103 (25.2%)	4 (57.1%)	99 (24.6%)	
**Total duration of resuscitation (minutes)**	55.0 [44.0;65.0]	30.0 [27.5;55.0]	55.0 [45.0;65.0]	0.076
**Duration of resuscitation after hospital arrival (min)**	6.00 [2.00;14.0]	5.00 [2.50;16.5]	6.50 [2.00;14.0]	0.992
**Duration of resuscitation before hospital arrival (min)**	44.0 [33.0;55.0]	27.0 [22.5;30.0]	45.0 [34.0;55.0]	0.004
**Use of automated chest compression device**	366 (89.5%)	7 (100%)	359 (89.3%)	1.000
**Any intervention during resuscitation**	34 (8.31%)	3 (42.9%)	31 (7.71%)	0.015
**Any supportive treatment during resuscitation**	70 (17.1%)	3 (42.9%)	67 (16.7%)	0.100
**Location of arrest:**				0.058
Place of residence	236 (57.7%)	1 (14.3%)	235 (58.5%)	
Public location	103 (25.2%)	4 (57.1%)	99 (24.6%)	
On hospital grounds	3 (0.73%)	0 (0.00%)	3 (0.75%)	
Other location	31 (7.58%)	1 (14.3%)	30 (7.46%)	
Missing	36 (8.80%)	1 (14.3%)	35 (8.71%)	
**Universal Termination of Resuscitation-rule:**				1.000
Terminate	124 (30.3%)	2 (28.6%)	122 (30.3%)	
Transport	247 (60.4%)	5 (71.4%)	242 (60.2%)	
Missing	38 (9.29%)	0 (0.00%)	38 (9.45%)	
**Any prehospital ROSC**	75 (18.3%)	3 (42.9%)	72 (17.9%)	0.119
**Any ROSC**	126 (30.8%)	7 (100%)	119 (29.6%)	<0.001
**Sustained ROSC**	53 (13.0%)	7 (100%)	46 (11.4%)	<0.001
**Admitted to intensive care or ward**	52 (12.7%)	7 (100%)	45 (11.2%)	<0.001
Intensive care	47 (11.5%)	7 (100%)	40 (9.95%)	
Regular ward	3 (0.73%)	0 (0.00%)	3 (0.75%)	
Unknown	2 (0.49%)	0 (0.00%)	2 (0.50%)	

Data are presented as mean (SD), absolute (relative) frequency or median [IQR]. Missing values (where present) are presented for all categorical variables. Missing data were omitted in the estimation of *P* values for all variables.

### The universal termination of resuscitation rule

Among the 409 patients, uTOR could be assessed in 371 cases (91%), the reason for missing cases was a lack of data on EMS witnessed status (*n* = 38). uTOR criteria for termination of resuscitation were fulfilled in 124 patients (30.3%). In 7 survivors, the uTOR recommended transport to hospital in 5 cases and termination of resuscitation for 2 patients. For the two false positive cases, the presumed cause of arrest was classified as non-cardiac and missing, respectively.

This corresponds to a positive predictive value (PPV) of 98.4% (95% CI 94.9–99.5%) and specificity of 71.4% for uTOR = terminate, had it been applied in the cohort with valid data (*n* = 371). With all missing values clustered among non-survivors, sensitivity analyses yielded no relevant changes (table S4).

### Additional treatment during resuscitation

A total of 34 patients (8.3%) were treated for a suspected reversible cause of arrest in the ED as per the 4H’s/4 T’s-classification (Table 2). Another 53 patients (13.0%) received supportive therapy only and 17 patients received both an intervention against a suspected reversible cause of arrest and supportive therapy (table S2).

**Table 2:** All interventions attempted during ongoing cardiopulmonary resuscitation.

Intervention	No. of attempts	No. of attempts followed by sustained ROSC	No. of attempts followed by survival to hospital discharge
**Advanced airway manoeuvres**	7	3	1
**Blood transfusion**	1	0	0
**Potassium correction**	1	0	0
**Rewarming after hypothermia**	1	0	0
**Coronary angiography**	7	3	2
**Percutaneous coronary intervention**	5	2	1
**Pericardial decompression**	9	1	0
**Intravenous thrombolysis**	4	1	0
**Pleural decompression**	1	0	0
**Antidote administration**	5	0	0
**Unique patients**	34	8	3

Individual patients might have received more than one intervention.

The most frequently attempted intervention was 9 cases of pericardial decompression due to suspected cardiac tamponade, one of which was followed by ROSC but not survival to hospital discharge. Coronary angiography was performed in 7 cases, with a subsequent percutaneous coronary intervention (PCI) attempted in 5 of them. Three patients eventually achieved sustained ROSC and 2 patients survived to hospital discharge (of whom 1 had a PCI). Advanced airway manoeuvres were performed in 7 patients, of whom one survived to hospital discharge.

Patients in whom an intervention was attempted were younger and had shorter durations of resuscitation prior to hospital arrival (Table 3). In-hospital resuscitation was significantly longer. There were no significant differences regarding prehospital non-sustained ROSC or uTOR-status between those receiving an intervention attempt versus those who did not. Among patients where an intervention attempt was made, more patients had sustained ROSC and survival was significantly higher.

**Table 3:** Baseline characteristics by any attempted intervention against a suspected reversible cause of arrest.

	Any intervention attempted		
	Yes *N* = 34	No *N* = 375	*P* Value
**Age (years)**	55.5 (17.7)	69.5 (15.7)	<0.001
**Sex: Male**	26 (76.5%)	255 (68.0%)	0.408
**Initial rhythm:**			0.486
Shockable	7 (20.6%)	126 (33.6%)	
Asystole	17 (50.0%)	164 (43.7%)	
PEA	9 (26.5%)	81 (21.6%)	
Unknown, no shocks delivered	0 (0.00%)	4 (1.07%)	
Missing	1 (2.94%)	0 (0.00%)	
**Defibrillation performed**	10 (29.4%)	173 (46.1%)	0.086
Missing	1 (2.94%)	11 (2.93%)	
**Witnessed**	24 (70.6%)	262 (69.9%)	1.000
Missing	0 (0.00%)	1 (0.27%)	
**EMS witnessed**	2 (5.88%)	36 (9.60%)	0.752
Missing	9 (26.5%)	69 (18.4%)	
**Presumed cardiac cause of arrest:**	12 (35.3%)	179 (47.7%)	0.406
Missing	11 (32.4%)	92 (24.5%)	
**Total duration of resuscitation (minutes)**	58.5 [45.0;77.5]	54.5 [43.0;63.0]	0.056
**Duration of resuscitation after hospital arrival (minutes)**	20.0 [10.0;30.0]	5.00 [2.00;11.2]	<0.001
**Duration of resuscitation before hospital arrival (minutes)**	38.0 [30.0;50.0]	45.0 [34.0;55.0]	0.049
**Location of arrest:**			0.181
Place of residence	16 (47.1%)	220 (58.7%)	
Public location	11 (32.4%)	92 (24.5%)	
On hospital grounds	1 (2.94%)	2 (0.53%)	
Other location	3 (8.82%)	28 (7.47%)	
Missing	3 (8.82%)	33 (8.80%)	
**Universal Termination of Resuscitation-rule:**			0.100
Terminate	15 (44.1%)	109 (29.1%)	
Transport	16 (47.1%)	231 (61.6%)	
Missing	3 (8.82%)	35 (9.33%)	
**Any prehospital ROSC**	6 (17.6%)	69 (18.4%)	1.000
**Any ROSC**	14 (41.2%)	112 (29.9%)	0.240
**Sustained ROSC**	8 (23.5%)	45 (12.0%)	0.064
**Admitted to ICU or ward**	8 (23.5%)	44 (11.7%)	0.059
**Discharged alive**	3 (8.82%)	4 (1.07%)	0.015

Data are presented as mean (SD), absolute (relative) frequency or median [IQR]. Missing values (where present) are presented for all categorical variables. Missing data were omitted in the estimation of *P* values for all variables.

## Discussion

The main findings of this study are that survival after OHCA is poor in patients transported to hospital with ongoing CPR and that in-hospital interventions targeting suspected reversible causes of arrest are rare. The few survivors had either a shockable rhythm or PEA as the presenting rhythm, while no patient with asystole survived to hospital discharge. The uTOR criteria, had they been applied, would have prevented transport of 122 non-survivors and of 2 patients who eventually survived.

The overall survival rate for patients arriving in the ED without sustained prehospital ROSC in the present study is poor and in line with data from West Midlands, UK (1.3%)^16^ and southern Ontario, Canada (2.3%),^3^ but significantly lower than the 20% survival rate for refractory OHCA reported from Copenhagen, Denmark.^17^ For a comparison to be meaningful, it has to be related to the overall proportion of patients with OHCA that is transported to hospital. The hospital-based registries used in the present study as well as the one by Yates et al. do not allow for a precise estimation of the transport frequency, but extrapolations from the national cardiac arrest registries of each country yield estimates of approximately 60% for our region and slightly less than 50% for West Midlands. In line with these estimates, the data reported by Drennan et al. for southern Ontario indicate that 54% of all patients with OHCA are transported to hospital. In this regard, Copenhagen stands out with a more restrictive approach where only 35% of all patients with OHCA are brought to hospital – of whom the clear majority (92%) had sustained field ROSC.^17^

While differences regarding EMS organisations make direct comparisons problematic, prehospital staffing and resources are comparable between our region, West Midlands and southern Ontario regarding an OHCA response. These services respond with ALS-capable units with over-the-phone physician consultation available when needed. Copenhagen, on the other hand, employs a system of emergency ambulances with BLS capabilities that are backed up by an on-site prehospital physician service in cases of OHCA.^17^ This unit is responsible for all decisions regarding transport to hospital or termination of resuscitation in the field. Interestingly, the extrapolated survival rate of OHCA patients without prehospital ROSC in our study is similar to that of southern Ontario as well as Copenhagen (1%), giving a rough indication that a more restrictive approach does not necessarily correspond to a loss of life. On the contrary, a recent analysis of a large north American registry indicates that patients transported with ongoing resuscitation efforts may fare worse than comparable patients in whom resuscitation was continued on scene.^18^ In this respect, it is concerning that 27 of the 159 patients (17%) who achieved sustained ROSC prior to hospital arrival in the present study did so after commencement of transport (table S3).

In the present study, only 21% of patients arriving in the ED without sustained ROSC received any treatment outside of the ACLS algorithm, and only 8% received a targeted intervention against a suspected cause of arrest. Given that prehospital resources in our region are fully ALS-capable, the value of hospital transfer is questionable for a majority of patients without field ROSC. Nevertheless, it cannot be ruled out that a select few patients might benefit from early transfer to hospital, but to identify them remains a challenge, as only 4 out of 409 (<1%) patients survived after having received any hospital-based therapy outside the ALS algorithm. Although the advent of eCPR might provide an additional treatment option for patients without field ROSC,^19^ this was not available in the current study setting. In addition, selection of patients who might benefit from this resource-intensive intervention remains a challenge,^20^ and is currently only recommended as a rescue therapy.^21^

In terms of its PPV, the uTOR performed reasonably well in the present study, with a point estimate of 98.4% among patients without sustained prehospital ROSC. This must, however, be interpreted with caution due to the low survival rate seen in this cohort, where simply classifying the entire cohort of 409 cases as “terminate” still would yield a PPV in excess of 98%. Thus, the poor specificity of 71% demonstrated here might have serious implications when applying the rule on cohorts with a higher expected survival rate. One reason for this observation might be the inclusion of patients with OHCA of all causes in the present study, as the uTOR originally was conceived for OHCA of a presumed cardiac cause only.^22^ In this context, two patients survived to hospital discharge despite meeting uTOR criteria for termination, corresponding to a 1.6% survival rate. Importantly, none of these two false positive cases had a presumed cardiac cause of arrest but the accuracy of such presumptions has been shown to vary.23., 24. Nonetheless, the survival rate presented here is higher than in previous studies of uTOR performance in cohorts of both presumed cardiac^3^ and non-cardiac^25^ causes of arrest and higher than the proposed 1% threshold for medical futility.^26^ As the false positive rate of the uTOR-criteria has been shown to increase with earlier application of the rule,^7^ the “load and go-strategy” encouraged in the setting of the present study might, at least in part, explain this finding. Indeed, one of the two patients that survived despite being classified as uTOR = terminate suffered a PEA arrest and was swiftly transported with ongoing resuscitation. Immediately after hospital arrival, he was found to have ROSC. The other survivor opted out of further data collection and analysis.

In summary, the findings of the present study do not support routine transportation of OHCA patients prior to achievement of field ROSC. In settings where transport with good quality CPR is feasible, this might however be a reasonable strategy for selected patients with a high suspicion of a reversible cause of arrest. In such cases, the decision to transport should be made swiftly, since both intervention attempts and survival are inversely associated with longer duration of prehospital resuscitation. Replicating the findings by Yates et al.,^16^ we found no survivors with an initial rhythm of asystole if field ROSC had not been achieved. Refraining from transportation of these patients would have prevented hospital transfer in 131 cases, equalling 32% of transported patients (data not shown). The poor specificity of the uTOR demonstrated in this setting supports the conclusion of a recent Consensus on Science with Treatment Recommendations (CoSTR) from the International Liaison Committee on Resuscitation (ILCOR) that clinical decision rules on termination of resuscitation need to be validated locally prior to implementation and used only as part of a holistic patient assessment.^27^

This retrospective analysis has multiple limitations. First, the use of a hospital-based registry limits generalisability of the results to the prehospital setting. Second, matching issues due to inconsistencies regarding social security numbers caused a significant amount of missing data for variables used to determine the uTOR-status and the presumed cause of arrest. A sensitivity analysis, however, yielded no clinically relevant changes due to all missing values being clustered among non-survivors. Moreover, one patient known to have survived after receiving supportive therapy opted out of further data collection and could therefore not be described and analysed in detail. In addition, lack of data on long-term neurological outcome is a limitation, and it must be noted that our results are not directly generalisable to settings where eCPR is utilised.

## Conclusion

Survival after OHCA where sustained ROSC is not achieved in the prehospital setting is rare and available in-hospital treatment resources are rarely utilised. The few survivors had either a shockable initial rhythm or presented with PEA, while no patient with asystole survived to hospital discharge. The uTOR criteria resulted in a positive predictive value of 98.4% for uTOR terminate but showed poor specificity.

## Ethical considerations

Ethical approval was granted by the Regional Ethical Review Board at Lund University (2016/995). Survivors received written information about the study and their right to opt out.

## Conflicts of interest

Simon Schmidbauer has received funding through a Swedish governmental grant for resident doctors pursuing research (ALF). Hans Friberg received governmental funding (ALF) and a grant from the Hans-Gabriel and Alice Trolle-Wachtmeister Medical Foundation. Gavin D. Perkins is co-chair in the International Liaison Committee on Resuscitation (ILCOR) and director of the European Resuscitation Council Research Net and has received funding through grants from the National Institute for Health Research (Health Service Delivery Research Programme and Applied Research). Authors EJY, CA, DB and HO have no conflicts of interest to declare.

## CRediT authorship contribution statement

**S. Schmidbauer:** Conceptualization, Methodology, Software, Formal analysis, Investigation, Data curation, Writing – original draft, Visualization. **EJ. Yates:** Conceptualization, Writing – review & editing. **C. Andréll:** Conceptualization, Writing – review & editing. **D. Bergström:** Investigation, Writing – original draft. **H. Olson:** Investigation, Data curation. **GD. Perkins:** Conceptualization, Writing – review & editing, Supervision. **H. Friberg:** Conceptualization, Methodology, Writing – original draft, Supervision, Project administration.

## References

[1] Dyson K., Brown S.P., May S. (2019). International variation in survival after out-of-hospital cardiac arrest: a validation study of the Utstein template. Resuscitation.

[2] Gräsner J.-T., Lefering R., Koster R.W. (2016). EuReCa ONE - 27 Nations, ONE Europe, ONE Registry: a prospective one month analysis of out-of-hospital cardiac arrest outcomes in 27 countries in Europe. Resuscitation.

[3] Drennan I.R., Lin S., Sidalak D.E., Morrison L.J. (2014). Survival rates in out-of-hospital cardiac arrest patients transported without prehospital return of spontaneous circulation: an observational cohort study. Resuscitation.

[4] Goto Y., Maeda T., Nakatsu-Goto Y. (2013). Neurological outcomes in patients transported to hospital without a prehospital return of spontaneous circulation after cardiac arrest. Crit Care.

[5] Adams B.D., Benger J. (2014). Should we take patients to hospital in cardiac arrest?. BMJ.

[6] Morrison L.J., Verbeek P.R., Zhan C., Kiss A., Allan K.S. (2009). Validation of a universal prehospital termination of resuscitation clinical prediction rule for advanced and basic life support providers. Resuscitation.

[7] Grunau B., Taylor J., Scheuermeyer F.X. (2017). External validation of the universal termination of resuscitation rule for out-of-hospital cardiac arrest in British Columbia. Ann Emerg Med.

[8] Drennan I.R., Case E., Verbeek P.R. (2017). A comparison of the universal TOR Guideline to the absence of prehospital ROSC and duration of resuscitation in predicting futility from out-of-hospital cardiac arrest. Resuscitation.

[9] Morrison L.J., Visentin L.M., Kiss A. (2006). Validation of a rule for termination of resuscitation in out-of-hospital cardiac arrest. N Engl J Med.

[10] Schmidbauer S., Herlitz J., Karlsson T., Axelsson C., Friberg H. (2017). Use of automated chest compression devices after out-of-hospital cardiac arrest in Sweden. Resuscitation.

[11] Divisionen MT och Prehospital Sjukvård. Vårdprogram PHAVIS Prehospital Hjärt Akutsjukvård i Skåne. 2018 [accessed 02/03/2021]. Available at: https://vardgivare.skane.se/siteassets/1.-vardriktlinjer/regionala-vardprogram---fillistning/phavis--final-27-apr-18.pdf.

[12] Møller T.P., Andréll C., Viereck S. (2016). Recognition of out-of-hospital cardiac arrest by medical dispatchers in emergency medical dispatch centres in two countries. Resuscitation.

[13] Soar J., Nolan J.P., Böttiger B.W. (2015). European Resuscitation Council Guidelines for Resuscitation 2015. Resuscitation.

[14] Perkins G.D., Jacobs I.G., Nadkarni V.M. (2015). Cardiac Arrest and Cardiopulmonary Resuscitation Outcome Reports: Update of the Utstein Resuscitation Registry Templates for Out-of-Hospital Cardiac Arrest: A Statement for Healthcare Professionals From a Task Force of the International Liaison Committee on Resuscitation (American Heart Association, European Resuscitation Council, Australian and New Zealand Council on Resuscitation, Heart and Stroke Foundation of Canada, InterAmerican Heart Foundation, Resuscitation Council of Southern Africa, Resuscitation Council of Asia); and the American Heart Association Emergency Cardiovascular Care Committee and the Council on Cardiopulmonary, Critical Care, Perioperative and Resuscitation. Circulation.

[15] Mercaldo N.D., Lau K.F., Zhou X.H. (2007). Confidence intervals for predictive values with an emphasis to case–control studies. Stat Med.

[16] Yates E.J., Schmidbauer S., Smyth A.M. (2018). Out-of-hospital cardiac arrest termination of resuscitation with ongoing CPR: an observational study. Resuscitation.

[17] Gregers E., Kjærgaard J., Lippert F. (2018). Refractory out-of-hospital cardiac arrest with ongoing cardiopulmonary resuscitation at hospital arrival – survival and neurological outcome without extracorporeal cardiopulmonary resuscitation. Crit Care.

[18] Grunau B., Kime N., Leroux B. (2020). Association of intra-arrest transport vs continued on-scene resuscitation with survival to hospital discharge among patients with out-of-hospital cardiac arrest. JAMA.

[19] Yannopoulos D., Bartos J., Raveendran G. (2020). Advanced reperfusion strategies for patients with out-of-hospital cardiac arrest and refractory ventricular fibrillation (ARREST): a phase 2, single centre, open-label, randomised controlled trial. The Lancet.

[20] Alm-Kruse K., Sørensen G., Osbakk S.A. (2021). Outcome in refractory out-of-hospital cardiac arrest before and after implementation of an ECPR protocol. Resuscitation.

[21] Perkins G.D., Gräsner J.-T., Semeraro F. (2021). European Resuscitation Council Guidelines 2021: Executive summary. Resuscitation.

[22] Verbeek P.R., Vermeulen M.J., Ali F.H. (2002). Derivation of a termination-of-resuscitation guideline for emergency medical technicians using automated external defibrillators. Acad Emerg Med.

[23] Kürkciyan I., Meron G., Behringer W. (1998). Accuracy and impact of presumed cause in patients with cardiac arrest. Circulation.

[24] Nashelsky M.B., Lawrence C.H. (2003). Accuracy of cause of death determination without forensic autopsy examination. Am J Forensic Med Pathol.

[25] Kashiura M., Hamabe Y., Akashi A. (2016). Applying the termination of resuscitation rules to out-of-hospital cardiac arrests of both cardiac and non-cardiac etiologies: a prospective cohort study. Crit Care.

[26] Schneiderman L.J., Jecker N.S., Jonsen A.R. (1990). Medical futility: its meaning and ethical implications. Ann Intern Med.

[27] Smyth M, Perkins G, Coppola A, et al. Prehospital termination of resuscitation (TOR) rules Draft Consensus on Science with Treatment Recommendations - on behalf of the International Liaison Committee on Resuscitation Education, Implementation and Teams Task Force; 2020 [accessed 18/03/2021]. Available at: http://ilcor.org.

