# Supplementary material for: Outcomes and interventions in patients transported to hospital with ongoing CPR after out-of-hospital cardiac arrest – An observational study
Source: Resusc Plus. 2021 Oct 16;8:100170. doi: 10.1016/j.resplu.2021.100170 (PMC8640866; doi:10.1016/j.resplu.2021.100170)
Supplement: Supplementary data 1 [file mmc1.pdf]

Supplement

Outcomes and interventions in patients transported to hospital with ongoing CPR after out-of-hospital cardiac arrest – an observational study by Schmidbauer, S et al.

Table S1 - Classification of interventions against suspected reversible causes of arrest

Reversible cause	Intervention
**Hypoxia**	Advanced airway manoeuvres†
**Hypovolemia**	Blood transfusion‡
**Hypo-/hyperkalemia**	Potassium correction§
**Hypothermia**	Rewarming manoeuvres including ECMO
**Thrombosis**	Coronary angiography
	Intravenous thrombolysis
**Tamponade**	Pericardial decompression
**Tension pneumothorax**	Pleural decompression
**Toxins**	Administration of any antidote

†Any stated airway manoeuvre targeted against either a suspected cause of arrest (such as foreign body removal) or factor sustaining cardiac arrest (such as removal of aspirated secretions preventing effective oxygenation). Routine endotracheal intubation was not classified as an advanced airway manoeuvre.

‡Due to a high frequency of crystalloid administration without any recorded indication, only transfusion of blood products was classified as a targeted intervention against hypovolemia.

§Any targeted therapy against either hypo- or hyperkalemia (such as potassium administration or glucose/insulin administration) or the negative effects thereof (such as calcium administration).

Table S2 - Overview of supportive therapies

Therapy	No. of attempts	No. of attempts followed by sustained ROSC	No. of attempts followed by survival to hospital discharge
Intravenous buffer administration	65	14	2
Infusion of inotropic or vasopressor agent	3	2	1
Medical therapy against bradycardia	2	2	0
Pacemaker (external or transvenous)	6	2	0
Repositioning of defibrillator pads	2	2	0
**Unique patients**	**69**	**17**	**2**

Individual patients might have received more than one therapy. One survivor who declined data collection on in-hospital therapies excluded.

Table S3 – Baseline characteristics of patients with sustained prehospital ROSC

	**All patients** N=158
**Age (years)**	69.2 (14.9)
**Sex:**	
Female	56 (35.4%)
Male	102 (64.6%)
**Initial rhythm:**	
Shockable	67 (42.9%)
Asystole	48 (30.8%)
PEA	32 (20.5%)
Unknown, no shocks delivered	9 (5.77%)
Missing	2 (1.27%)
**Defibrillation performed**	72 (50.7%)
Missing	16 (10.1%)
**Witnessed**	128 (81.0%)
**EMS witnessed**	21 (16.8%)
Missing	33 (20.9%)
**Presumed cause of arrest:**	
Cardiac	59 (53.2%)
Non-cardiac	52 (46.8%)
Missing	47 (29.7%)
**Total duration of resuscitation (minutes)**	15.0 [10.0;27.0]
**Duration of resusciation after hospital arrival (minutes)**	0.00 [0.00;0.00]
**Duration of resuscitation before hospital arrival (minutes)**	15.0 [8.25;26.0]
**Use of automated chest compression device**	110 (70.1%)
Missing	*1 (0.63%)*
**Location of arrest:**	
Place of residence	91 (60.7%)
Public location	35 (23.3%)
On hospital grounds	2 (1.33%)
Other location	22 (14.7%)
Missing	8 (5.06%)
**Location of sustained ROSC:**	
Prehospital, on scene	129 (81.6%)
Prehospital, in ambulance	27 (17.1%)
Prehospital, unknown location	2 (1.27%)
**Admitted to intensive care or ward:**	
Intensive care	105 (66.5%)
Regular ward	8 (5.06%)
Admitted, level of care unknown	26 (16.5%)
Not admitted	19 (12.0%)
**Discharged alive**	73 (46.2%)

Data are presented as mean (SD), absolute (relative) frequency or median [IQR]. Missing values (where present) are presented for all categorical variables.

Table S4 – Sensitivity analysis: Diagnostic performance of the universal termination of resuscitation rule

Cohort	PPV (%)	95 % CI (%)	Specificity (%)
**Valid data (n = 371)**	98.4	94.9-99.5	71.4
**Best case (n = 409)**[†]	98.8	96.1-99.6	71.4
**Worst case (n = 409)**[‡]	98.4	94.9-99.5	71.4

PPV**:** Positive predictive value. [†]All missing cases imputated as uTOR = terminate (i.e. true positives). [‡]All missing cases imputated as uTOR = transport (i.e. false negatives)
